# Detecting Intraocular Pressure Harmonic Frequencies in Glaucoma and the Sampling Targets Needed to Identify Them With Self-Tonometry

**DOI:** 10.1167/iovs.67.12.37

**Published:** 2026-10-21

**Authors:** William H. Morgan, Chiau-Ni (Charlene) Chiu, Andrew Turpin, Danail Obreschkow, Allison M. McKendrick

**Affiliations:** 1 Lions Eye Institute, University of Western Australia, Royal Perth Hospital, Perth, Western, Australia; 2 School of Health and Clinical Sciences, University of Western Australia, Perth, Western, Australia; 3 School of Population Health, Curtin University, Perth, Western, Australia; 4 International Centre for Radio Astronomy Research, The University of Western Australia, Crawley, Western, Australia; 5 International Space Centre, The University of Western Australia, Crawley, Western, Australia

**Keywords:** intraocular pressure (IOP), circadian rhythm, home tonometry, iCare HOME, harmonic analysis, Lomb–Scargle periodogram (LSP), generalized additive model (GAM), sampling design, glaucoma

## Abstract

**Purpose:**

The purpose of this study was to determine the detectability and occurrence of circadian and ultradian rhythms in patient-acquired intraocular pressure (IOP) measurements with sampling targets for reliable harmonic detection.

**Methods:**

A multi-harmonic non-uniform discrete Fourier transform (NDFT), with bootstrap amplitude confidence intervals and independent Lomb–Scargle periodogram (LSP) confirmation, was applied to 286 home-tonometry monitoring blocks from 137 eyes of 69 patients with glaucoma. Integer harmonic frequencies (F) from 1 to 10 cycles per day were tested. Characterized frequency was defined when noise-threshold significance, amplitude lower 95% confidence bound >0, and phase 95% confidence interval met prespecified criteria. Detection probability was modeled by a logistic generalized additive model (GAM) accounting for total observation count, clock-hour coverage, and the longest sparse-data gap.

**Results:**

The 24-hour rhythm (F1) was characterized in 35.7% of blocks; at least one of F1, F2, F3, or F4 was characterized in 44.8% (128/286). Of these 128 blocks, 34% (43) had 2 or more harmonics simultaneously characterized and 7% (9) had 3 or more. Detected periodicities were almost exclusively integer harmonics. Non-integer LSP peaks arose in only 3.1% of blocks (9/286). Where 15 or more clock-hours contained at least 2 observations and no more than 2 consecutive clock-hours contained one or none, approximately 100 to 130 observations over 4 weeks gave a model-predicted detection probability of 75% to 85%, rising to 90% at approximately 150 observations.

**Conclusions:**

One or more characterizable 24-hour integer IOP harmonic rhythms could be detected with an appropriate self-tonometry protocol in nearly half of treated patients with glaucoma.

Elevated intraocular pressure (IOP) is the major risk factor for glaucoma development and progression.^1^ IOP varies systematically across the 24-hour cycle, and both fluctuation and elevated peak pressures may contribute to glaucomatous damage.^2^^,^^3^ Quasi-continuous monitoring of the diurnal curve has been an aspiration of clinical glaucoma assessment with suggestions that characterization of IOP fluctuation may identify another modifiable risk factor for glaucoma progression.^4^^–^^7^ This has been previously attempted, however, in-office tonometry captures only a narrow interval, in-hospital 24-hour monitoring is resource-intensive, and contact lens sensors report only arbitrary units.^8^^–^^11^ Patient-operated rebound tonometry with the iCare HOME (iCare Oy, Vantaa, Finland)^7^^,^^12^^–^^14^ makes weeks to months of self-recording possible, but sampling is patient-determined and therefore irregular. Single-cosine cosinor methods,^15^^,^^16^ dual-harmonic regression without per-component significance testing,^17^^,^^18^ and simple peak-and-trough description^19^ are inadequate for quantifying and confirming significant harmonics beyond the 24-hour fundamental.

Other physiological systems with mixed central and peripheral drivers show the same limitation of single-sinusoid models. Ambulatory blood pressure carries a significant 12-hour harmonic in approximately ⅔ of adults,^20^^,^^21^ and cortisol and glucose each combine a 24-hour rhythm with sub-daily structure.^22^^,^^23^ For irregularly sampled series, the standard tool is the Lomb–Scargle periodogram (LSP),^24^^–^^27^ which fits sinusoids at the true observation times but pays a multiple-comparison cost across its scan window. Where the rhythms of interest are integer harmonics of a known 24-hour fundamental, a least-squares Fourier fit confined to those frequencies is more powerful and yields a phase estimate for each component.^28^

This report is primarily a methodological development. Its purpose is to establish how IOP fluctuation may be resolved into its underlying frequencies, and to define the sampling conditions under which that resolution is reliable. The clinical motivation is twofold. Knowledge of the phase of an individual’s rhythm may allow beneficial treatment timing adjustment, and a continuous description of the pressure profile allows pressure-over-time and fluctuation metrics to be derived. Our findings describe a treated glaucoma population and are not offered as normative.

We put four questions to a large self-tonometry dataset, and they are taken up in this order in the Results and answered together at the head of the Discussion. How often is a 24-hour IOP rhythm accompanied by characterized higher order harmonics? Do the multi-harmonic Fourier method and the Lomb-Scargle periodogram differ in sensitivity? Are the constituent periodicities of IOP mainly integer or non-integer multiples of the primary 24-hour rhythm? How many self-measurements, and what distribution of them, are required to characterize these rhythms?

## Methods

### Study Population and Tonometry

The study was approved by the University of Western Australia Human Research Ethics Committee (2025_ET000031 and 2025_ET000351) and adhered to the Declaration of Helsinki. Informed consent was obtained from all participants. The dataset comprised 286 home-tonometry monitoring blocks from 137 eyes of 69 patients managed for glaucoma or ocular hypertension at the Lions Eye Institute, Perth, Australia. All measurements were acquired by patients at home using the iCare HOME (TA022) or iCare HOME2 rebound tonometer according to the manufacturer’s instructions; the HOME2 also records posture. Patients were trained in the sitting posture and certified for independent use when a first home measurement fell within ±5 millimeters of mercury (mm Hg) of contemporaneous Goldmann applanation tonometry. Measurement frequency was patient-determined, with guidance of two to four observations per day.

### Monitoring Blocks

Each patient record was partitioned into break-separated blocks by inserting a structural break wherever 10 or more days separated consecutive measurement days. Within a block, any 14-day window containing fewer than 20 observations was treated as sparse, and days lying inside such a window that carried 2 or fewer observations were excluded from the harmonic fit. Blocks were analyzed independently and empty inter-block intervals discarded.

### Harmonic Analysis Pipeline

Each block was processed by a three-stage pipeline implemented in R software. The stages were Fourier decomposition, refinement of harmonic modes, and an independent Lomb–Scargle scan.

### Fourier Decomposition Stage

Each block was decomposed into 10 integer harmonic frequencies from 1 to 10 cycles per day (periods 24, 12, 8, 6, 4.8, 4, 3.4, 3, 2.7, and 2.4 hours) by a non-uniform discrete Fourier transform, using a modification of the routine *ndft* of the R software package *cooltools*. Power was computed at each candidate frequency directly from the observations at their true irregular times; the data were never interpolated, binned, or resampled onto a uniform time mesh. Observed power was judged against the power the same measurements would produce if they contained no rhythm at all. Both quantities are periodogram ordinates evaluated at a single frequency rather than spectral densities, so both carry units of mm Hg² and their ratio is dimensionless. The reference level can be written down exactly. It is the number of observations multiplied by the variance of the pressures. That is the mean power expected at one tested frequency and not power accumulated across the spectrum, and it takes the same value at every frequency tested, because random scatter favors no one periodicity over another. The comparison is therefore made frequency by frequency. Each observed peak was divided by that reference level to give a signal-to-noise ratio, and because that ratio has a known distribution when no rhythm is present, the probability that scatter alone would produce a peak that large can be read directly from it. A harmonic was retained as significant when that probability fell below 0.001, the 99.9th centile of the null distribution, equivalent to a ratio above 6.91 (Supplementary Section S1.2). A 5%-of-frequency tolerance allowed a peak at a non-exact frequency to be deemed integer (Supplementary Section S1).

### Refinement Stage

All peaks passing the noise-threshold criterion were re-fitted simultaneously by least squares at their exact integer frequencies, from which the amplitude (mm Hg) and phase (hours) of each retained component were re-estimated. The fitted model is a mean level plus one cosine for each retained harmonic k, of the form A cos(2πkt/24 + φ) with t measured in hours from midnight, so it is the cosinor model^15^^,^^16^ generalized to several harmonics estimated simultaneously. Phase is reported throughout as the acrophase, the clock time at which a component reaches its maximum, referenced to midnight, and harmonic k reaches that maximum again every 24/k hours. Because spectral power belonging to one underlying rhythm spreads across neighboring frequencies, more than one adjacent peak may exceed the noise criterion near a single true periodicity. Such peaks are neither summed over a band nor discarded. A peak inside the tolerance window of a harmonic is taken as evidence that the harmonic is present, and the joint re-fit at the exact integer frequency then recovers its power in a single component, whereas a peak outside every window plays no part in the integer model and is considered only in the Lomb–Scargle scan (Supplementary Section S1). Confidence intervals on amplitude and phase were obtained by nonparametric bootstrap. A frequency was classified as characterized only when three criteria were met. These were noise-threshold significance, a bootstrap lower 95% confidence bound on amplitude above zero, and a bootstrap 95% confidence interval on phase narrower than 1/(2f) of a cycle at frequency f cycles per day, so that phase precision is required in proportion to how often a component recurs within the day. For integer harmonic k, that is an acrophase interval narrower than 24/(2k²) hours. This is a more demanding rule at the higher harmonics than a flat half-cycle criterion would be, and it makes the counts reported here conservative (Supplementary Section S3.1). The equivalence of this fit to a least-squares Lomb–Scargle analysis, and the consequent inapplicability of the classical Nyquist limit,^24^^–^^28^ are set out in Supplementary Section S3.1.

### Lomb–Scargle Scan

The generalized LSP^24^^–^^26^^,^^28^ was computed independently for each block using the *lomb* R software package, with the false-alarm probability calibrated by the method of Baluev.^29^ The scan ran from 0.1 to 10.5 cycles per day, that is from a period of 10 days down to a period of 2.3 hours, extending half a harmonic beyond F10 at its upper end. The same 5%-of-frequency tolerance classified peaks as integer or non-integer, so that the integer-harmonic counts of the 2 methods are comparable, and peaks outside the ±5% windows around all of F1 to F10 were treated as non-integer. An integer harmonic was recorded as Lomb–Scargle confirmed only when the false-alarm probability at that harmonic fell below 0.001 and the same amplitude and phase precision criteria used for characterization were also met, evaluated at the same exact integer frequency. Non-integer peaks were refitted on the integer grid alongside the harmonics of their own block, as described in the Results (Supplementary Section S2.2).

### Rationale for an Integer-Harmonic Framework

The two methods are complementary. The Fourier stage evaluates power on a prescribed grid of integer multiples of one cycle per day, carrying greater statistical power at those frequencies but limited sensitivity between them; the Lomb–Scargle scans continuously and detects whether a non-integer rhythm may be present. Because the significant periodicities proved to lie overwhelmingly on the integer grid (Results), the integer-harmonic decomposition was adopted for the principal analysis, and any apparent non-integer harmonic feature in a Fourier spectrum is adjudicated on the LSP.

### Sampling-Coverage Metrics

For each block, we recorded the total number of observations together with three descriptors of how they were distributed around the clock. The “observations-per-window minimum” is the smallest number of observations taken in any of the 8 non-overlapping 3-hour clock windows. The “clock-hour spread” is the number of the 24 clock-hours containing at least 2 observations. The “longest sparse gap” is the longest unbroken run of clock-hours each containing one or no observation.

### Statistical Analysis

The probability of characterizing at least one of F1 to F4 was modeled by a logistic generalized additive model (GAM) with a two-dimensional tensor smooth on the clock-hour spread and longest sparse gap, plus a smooth on total observations, implemented in the R software package mgcv^31^ with the binomial family and REML smoothness selection. A GAM was preferred to parametric logistic regression because the coverage predictors were strongly non-normal. Model specification and comparison are given in Supplementary Section S3.2. Per-block parameters across detection classes were compared by Kruskal–Wallis tests with Bonferroni-corrected pairwise Mann–Whitney *U* tests (Supplementary Section S4), and McNemar’s test with Cohen’s kappa was used for paired Fourier/Lomb–Scargle comparisons. Median and interquartile range (IQR) are reported throughout; significance is at *P* < 0.05. All analyses were performed in R software (version 4.3).^30^ Claude Opus 5.0 (Anthropic, San Francisco, CA, USA) was used on August 16, 2026, to modify the R code producing the figures.

## Results

### Dataset Characteristics

The 286 monitoring blocks, comprising 28,781 measurements, were derived from 137 eyes of 69 patients (Table 1). Primary open-angle glaucoma was present in 94% of eyes and the remainder had ocular hypertension; all participants were on medical therapy or had undergone previous drainage surgery. Posture was recorded in 89% of measurements, 99.84% of which were sitting. Median block duration was 22.4 days (range = 0.3–1078.0), and the number of observations per block was right-skewed.

**Table 1.** Summary Characteristics of the 286 iCare HOME Monitoring Blocks

Parameter	Value
Total monitoring blocks	286
Unique patients	69
Females, *n* (%)	38 (55)
Age, y, mean ± SD	66.0 ± 11.4
Visual field mean deviation, dB, mean ± SD	−4.59 ± 4.94
Unique patient–eye combinations	137
Right eye (OD) blocks	144 (50.3%)
Left eye (OS) blocks	142 (49.7%)
Block duration: median (range)	22.4 days (0.3–1078.0)
Total observations per block: median (range)	74 (3–2407)
Mean observations per block	100.6
Blocks with N_obs < 40	85 (29.7%)
Blocks with N_obs 40–70	47 (16.4%)
Blocks with N_obs 70–100	58 (20.3%)
Blocks with N_obs 100–130	57 (19.9%)
Blocks with N_obs 130–160	14 (4.9%)
Blocks with N_obs > 160	25 (8.7%)

N_obs, total IOP observations per block.

### Worked Example of the Pipeline

Figure 1 shows the complete pipeline output for one representative monitoring block (right eye, 131 observations across 35 days), presented first so that the summary results may be read against a concrete example. The recording contained an early 6-day calendar gap followed by a dense run of self-measurements. Despite that gap, 19 of the 24 clock-hours contained at least 2 observations with no consecutive sparse clock-hours, and both the 24-hour and 12-hour harmonics met all characterization criteria. The fitted curve shows 2 pressure peaks and 2 troughs per 24-hour cycle, a pattern a single cosine would average into one intermediate peak. The block also illustrates the treatment of non-integer features. A Fourier peak at 1.38 cycles per day exceeded the noise threshold and was in fact taller than the 12-hour harmonic, but it lies well outside the tolerance window of every integer harmonic, so it was never tested for characterization, and it did not reach significance on the LSP either.

**Figure 1.**
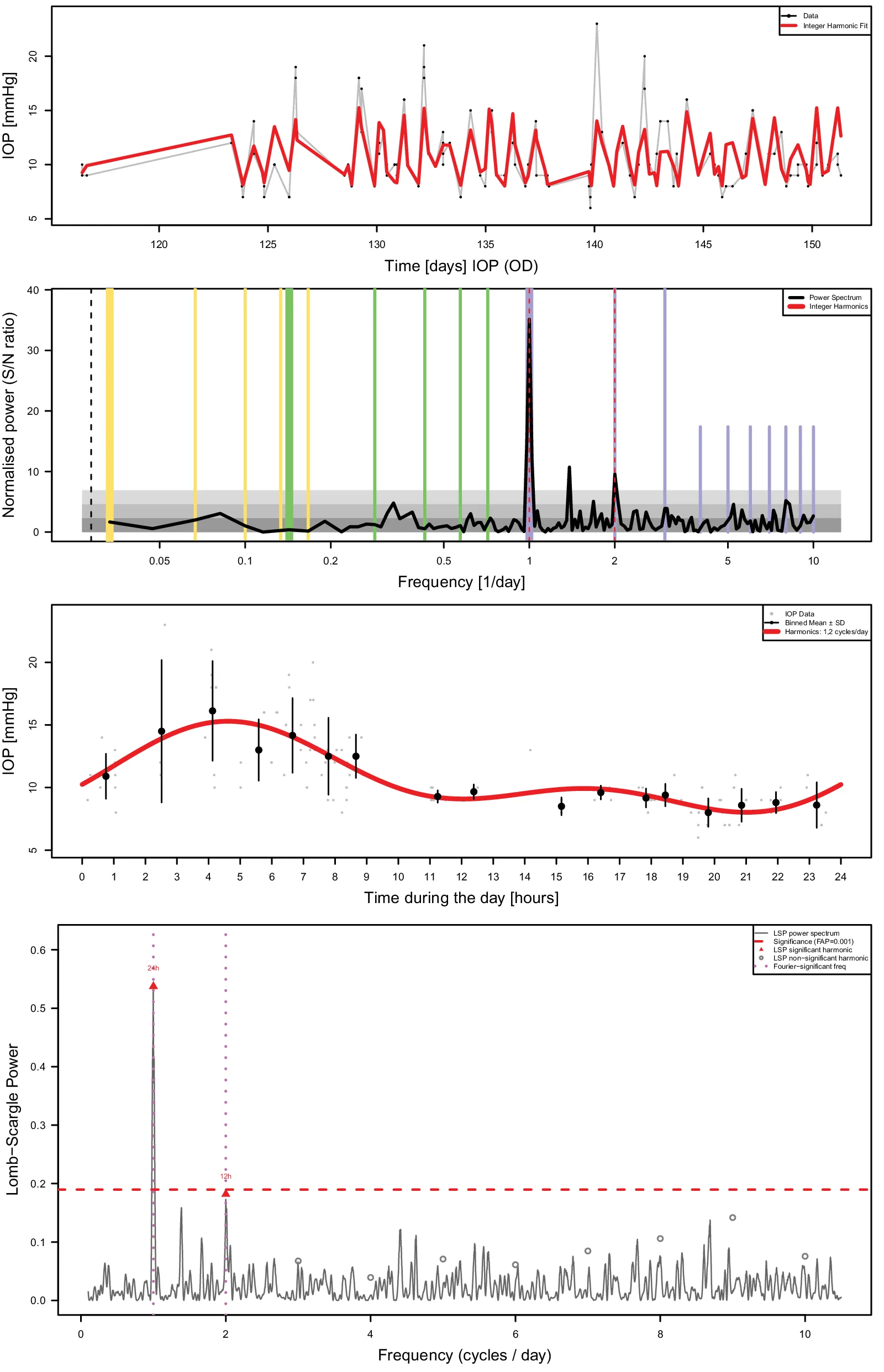
Worked example of the analysis output for one iCare HOME monitoring block of 131 observations over 35 days (right eye), illustrating every stage of the pipeline. The *top panel* shows the raw IOP time series (*grey*) against calendar time, with the fitted integer-harmonic model (*red*) comprising 24-hour and 12-hour components. The second panel shows the Fourier power spectrum, plotted as the ratio of observed power to the power expected in the absence of any rhythm, which is the number of observations multiplied by the variance of the measured pressures. Both are periodogram ordinates at a single frequency in mm Hg², so the plotted ratio is dimensionless and the axis is labeled as a normalized power, or signal-to-noise ratio. The nested *grey* bands are the 90th, 99th, and 99.9th centiles of that null distribution, lying at ratios of 2.30, 4.61, and 6.91, and a peak is significant only when it rises above the palest band (99.9th centile, *P* > 0.999). *Violet* bars mark the integer-harmonic grid from 1 to 10 cycles per day, whereas *yellow* and *green* bars mark monthly and weekly frequencies and their harmonics. Two integer peaks, at 1 and 2 cycles per day, reached characterization. The taller feature lying between them, at 1.38 cycles per day with a ratio of 10.7 against 9.6 at 2 cycles per day, sits 0.38 and 0.62 cycles per day away from those harmonics and therefore falls outside every integer tolerance window, so it is never a candidate for characterization; nor is it confirmed in the *bottom panel*, where it reaches a Lomb–Scargle power of 0.159 against the 0.190 required at a false-alarm probability of 0.001. The third panel shows the same observations folded onto a 24-hour clock face, with binned mean ± SD and the two-harmonic fitted curve. The *bottom panel* shows the generalized LSP of the same block, in which the 24-hour and 12-hour harmonics are flagged as significant (*red triangles*) against the false-alarm-probability threshold (FAP = 0.001; *red dashed line*), and the *orchid dotted line* marks the frequency of the corresponding Fourier-significant peak. Residual RMS of the fit is 2.19 mm Hg.

### Detection Rates Across the Harmonic Frequency Series

The 24-hour rhythm (F1) was characterized in 102 of 286 blocks (35.7%; Table 2; Fig. 2). The 12-hour (F2), 8-hour (F3) and 6-hour (F4) rhythms were characterized in 41 (14.3%), 21 (7.3%), and 16 (5.6%) blocks, respectively. Characterization at periods shorter than 6 hours was rare, reaching at most 0.7% of blocks (see Fig. 2). At least one of F1 to F4 was characterized in 128 of 286 blocks (44.8%).

**Table 2.** Detection Counts for all 10 Integer Harmonic Frequencies Across the 286 Monitoring Blocks

Frequency	Period, h	Fourier-Significant, *n*	Characterized, *n*	Lomb–Scargle Confirmed, *n*	% Characterized
Freq 1	24	107	102	91	35.7
Freq 2	12	53	41	32	14.3
Freq 3	8	33	21	10	7.3
Freq 4	6	29	16	5	5.6
Freq 5	4.8	5	1	0	0.3
Freq 6	4	5	2	1	0.7
Freq 7	3.4	5	2	0	0.7
Freq 8	3	1	0	0	0.0
Freq 9	2.7	0	0	0	0.0
Freq 10	2.4	0	0	0	0.0

Fourier-significant indicates noise-threshold P > 0.999; characterized indicates that all three quality criteria (noise-threshold significance, amplitude lower 95% confidence interval > 0, and an acrophase 95% confidence interval narrower than 24/(2k²) hours at harmonic k) were met; Lomb–Scargle confirmed indicates an independent Lomb–Scargle false-alarm probability < 0.001 at that harmonic together with the same amplitude and phase precision criteria. The two right-hand columns differ in how detection was established.

**Figure 2.**
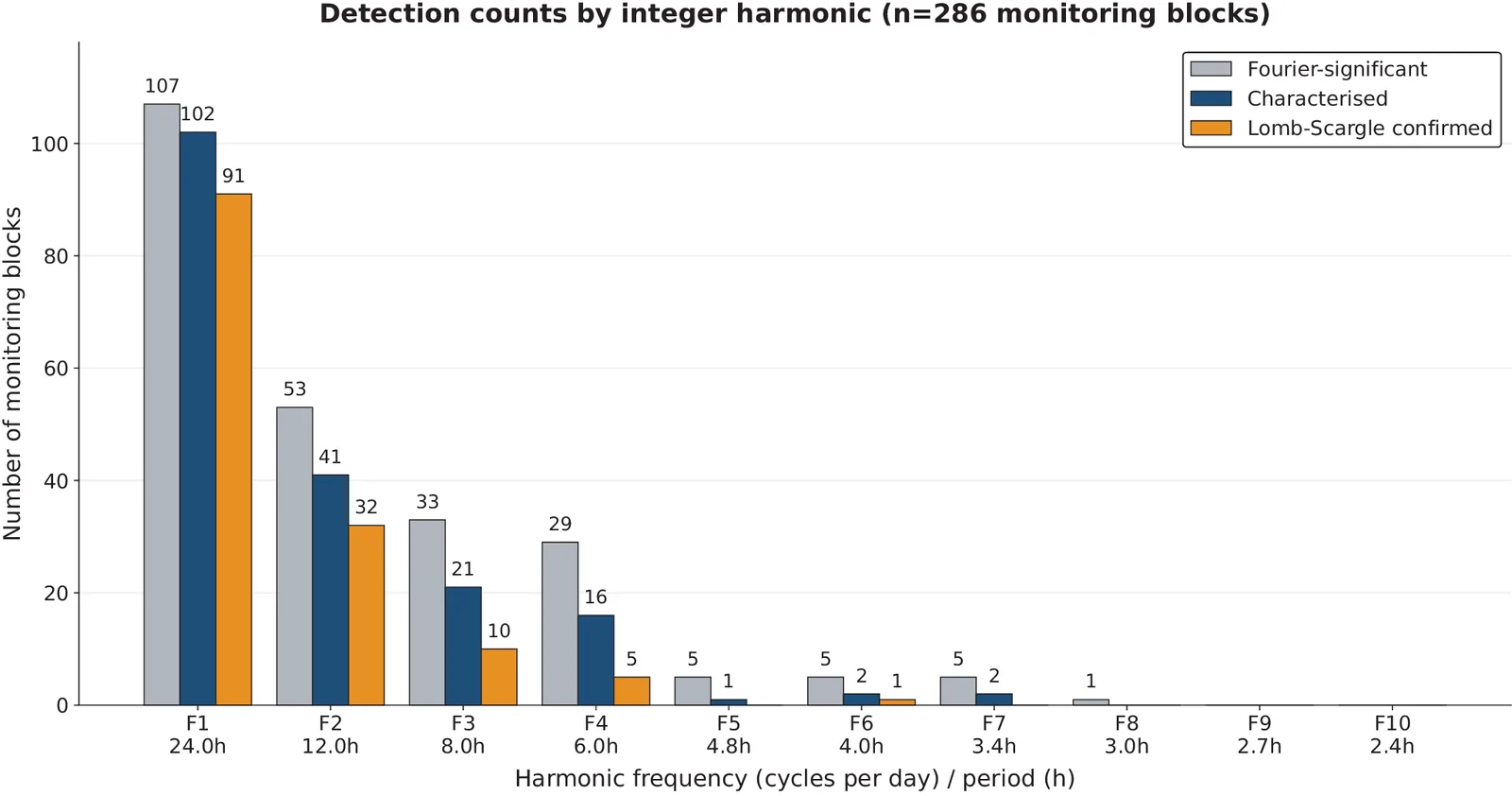
Detection counts for each integer harmonic frequency from 1 cycle per day (24-hour period) through 10 cycles per day (2.4-hour period) across the 286 monitoring blocks. Three categories are shown. Fourier-significant denotes noise-threshold *P* > 0.999. Characterized denotes Fourier-significant peaks that additionally met the amplitude and phase quality criteria. Lomb–Scargle confirmed denotes a false-alarm probability below 0.001 under the shared 5%-of-frequency integer rule, together with the same amplitude and phase criteria.

### Prevalence of Multi-Harmonic Characterization

Forty-three of 286 blocks (15.0%) had 2 or more of F1 to F4 simultaneously characterized and 9 (3.1%) had 3 or more. Of the 102 F1-characterized blocks, 39 (38.2%) also had at least one higher harmonic characterized, comprising 31 (30.4%) with F2, 10 (9.8%) with F3, and 7 (6.9%) with F4.

### Comparison of the Two Methods

Across F1 to F4, the 2 methods agreed substantially (pooled Cohen’s κ = 0.80; across all 10 harmonics, overall agreement 98.0% and pooled κ = 0.81; see Table 2; Fig. 2). The Fourier method characterized more blocks than the Lomb–Scargle confirmed at every harmonic, and the margin widened with harmonic order. At F1, the counts were 102 vs. 91, with 15 blocks unique to the Fourier method and 4 unique to the Lomb–Scargle (κ = 0.85; McNemar *P* = 0.022). At F2, they were 41 vs. 32 (11 and 2 unique; κ = 0.80; *P* = 0.027), at F3, they were 21 vs. 10 (11 and 0 unique; κ = 0.63; *P* = 0.003), and at F4, they were 16 vs. 5 (11 and 0 unique; κ = 0.46; *P* = 0.003). Pooled across all 10 harmonics there were 52 characterized-only against 6 Lomb–Scargle-only detections (McNemar χ² = 34.9, *P* < 0.001). Compared at the detection stage alone, before any amplitude or phase precision is required, the same direction holds (κ = 0.82, 0.66, 0.44, and 0.27 at F1–F4).

### Integer Versus Non-integer Harmonics

Detected rhythms were almost exclusively integer harmonics of the 24-hour fundamental. The LSP identified 15 non-integer peaks across 9 of 286 blocks (3.1%) from 6 subjects (Fig. 3; Table 3). In the remaining 277 blocks, every above-threshold Lomb–Scargle peak coincided with an integer harmonic within the ±5% tolerance. Each detected non-integer peak was then re-fitted jointly with the integer harmonics of its own block and put through the same amplitude and phase criteria used to characterize those harmonics. Nine of the 15 met both criteria and 6 did not (see Table 3). The confirmed peaks had periods between 3.64 and 69.20 hours and arose in 5 of the 6 subjects. We then asked whether any 2 of these non-integer peaks agreed with each other, applying to them the same 5% rule used to decide whether a peak lies on the integer grid. Only one pair did. SUBJ04 and SUBJ05 each carried a confirmed peak at 0.947 cycles per day, the 2 agreeing to within 0.07%. That frequency sits 0.053 cycles per day from the 24-hour fundamental, just 0.003 cycles per day outside the 0.05 window that would have classified it as F1. No other pair came within 5% of each other, the closest being 19.8% apart, so apart from this single near-miss on the daily rhythm no non-integer frequency was shared by 2 blocks or by 2 subjects. The 4 near-integer peaks (Δ ≤ 0.20 c/d) are SUBJ04 and SUBJ05 at 0.95 c/d, SUBJ03 at 1.17 c/d, and SUBJ01 at 1.81 c/d. Their interpretation, together with the 2 confirmed peaks whose period exceeds 48 hours, is given in Supplementary Section S5.

**Figure 3.**
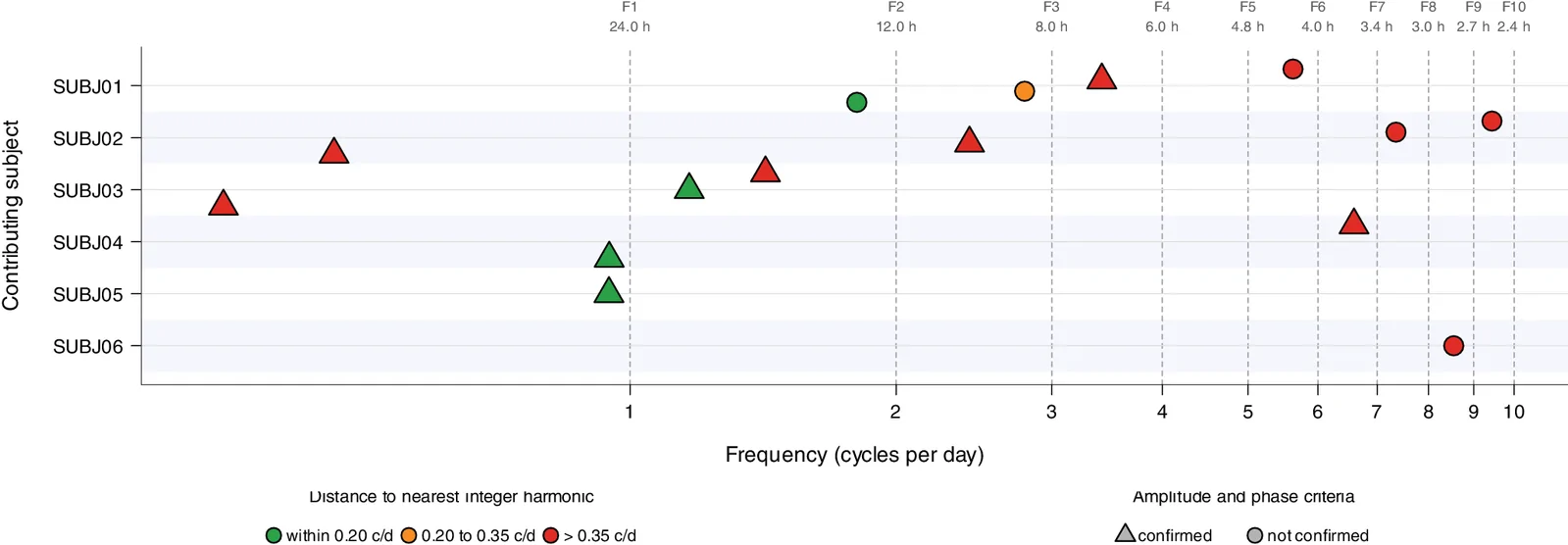
Distribution of the 15 non-integer Lomb–Scargle peaks across the frequency axis under the shared 5%-of-frequency integer rule. Each of the six contributing subjects occupies a separate horizontal lane (SUBJ01–SUBJ06, numbered as in Table 3). Each marker is one peak plotted at its cycles-per-day position within that subject’s lane, and peaks are offset vertically within a lane so that all 15 are individually visible. Integer-harmonic landmarks F1 to F10 are marked by *vertical dashed lines* labeled with their period in hours. Marker color encodes absolute distance to the nearest integer harmonic. *Green* marks a peak within 0.20 c/d, *orange* one between 0.20 and 0.35 c/d, and *red* one further than 0.35 c/d. Marker shape encodes the outcome of the joint refit. *Triangles* mark the nine peaks that met the same amplitude and phase criteria applied to the integer harmonics (confirmed), and circles the six that did not. The only two peaks sharing a frequency are the SUBJ04 and SUBJ05 markers close to F1.

**Table 3.** Per-Subject Distribution of the 15 Non-Integer Lomb–Scargle Peaks Across 9 of 286 Monitoring Blocks, Separated Into Those Confirmed and Those not Confirmed When Re-Fitted Jointly With the Integer Harmonics of the Same Block Under the Same Amplitude and Phase Criteria

Subject	Status	*n* Peaks	Periods, h	Frequencies, c/d	Nearest Integer Harmonic, Δ, c/d
SUBJ01	Confirmed	1	7.02	3.417	F3 (0.42)
SUBJ01	Not confirmed	3	13.29, 8.59, 4.27	1.805, 2.795, 5.622	F2 (0.20); F3 (0.21); F6 (0.38)
SUBJ02	Confirmed	2	51.88, 9.91	0.463, 2.421	F1 (0.54); F2 (0.42)
SUBJ02	Not confirmed	2	3.26, 2.54	7.351, 9.439	F7 (0.35); F9 (0.44)
SUBJ03	Confirmed	3	69.20, 20.57, 16.87	0.347, 1.167, 1.423	F1 (0.65); F1 (0.17); F1 (0.42)
SUBJ04	Confirmed	2	25.34, 3.64	0.947, 6.590	F1 (0.05); F7 (0.41)
SUBJ05	Confirmed	1	25.35	0.947	F1 (0.05)
SUBJ06	Not confirmed	1	2.81	8.545	F9 (0.46)

Δ is the absolute distance in cycles per day from the nearest integer harmonic F1–F10; all 15 peaks lie outside the harmonic-specific 5% acceptance window that would otherwise classify them as integer.

### Distribution of Measurements Across the Clock Face

The distribution of self-measurement times was examined across the whole dataset because harmonic detection depends upon the measurement times across the measurement period, here, the 24-hour cycle (Fig. 4). Sampling was heavily weighted toward waking hours. Across the six 3-hour windows spanning 06:00 to 24:00, a mean of 89.3% of blocks contained at least one observation, against 25.3% across the 2 overnight windows, a ratio of approximately 3.5 to 1. That proportion, which is what we mean by the coverage of a window, formed a broad plateau within waking hours, highest between 09:00 and 12:00 (93.4% of blocks) and lowest between 18:00 and 21:00 (84.6%). The same profile persisted at depths of 2, 3, and 5 observations per window, at which the day-to-night ratio widened to 4.7, 5.4, and 7.7.

**Figure 4.**
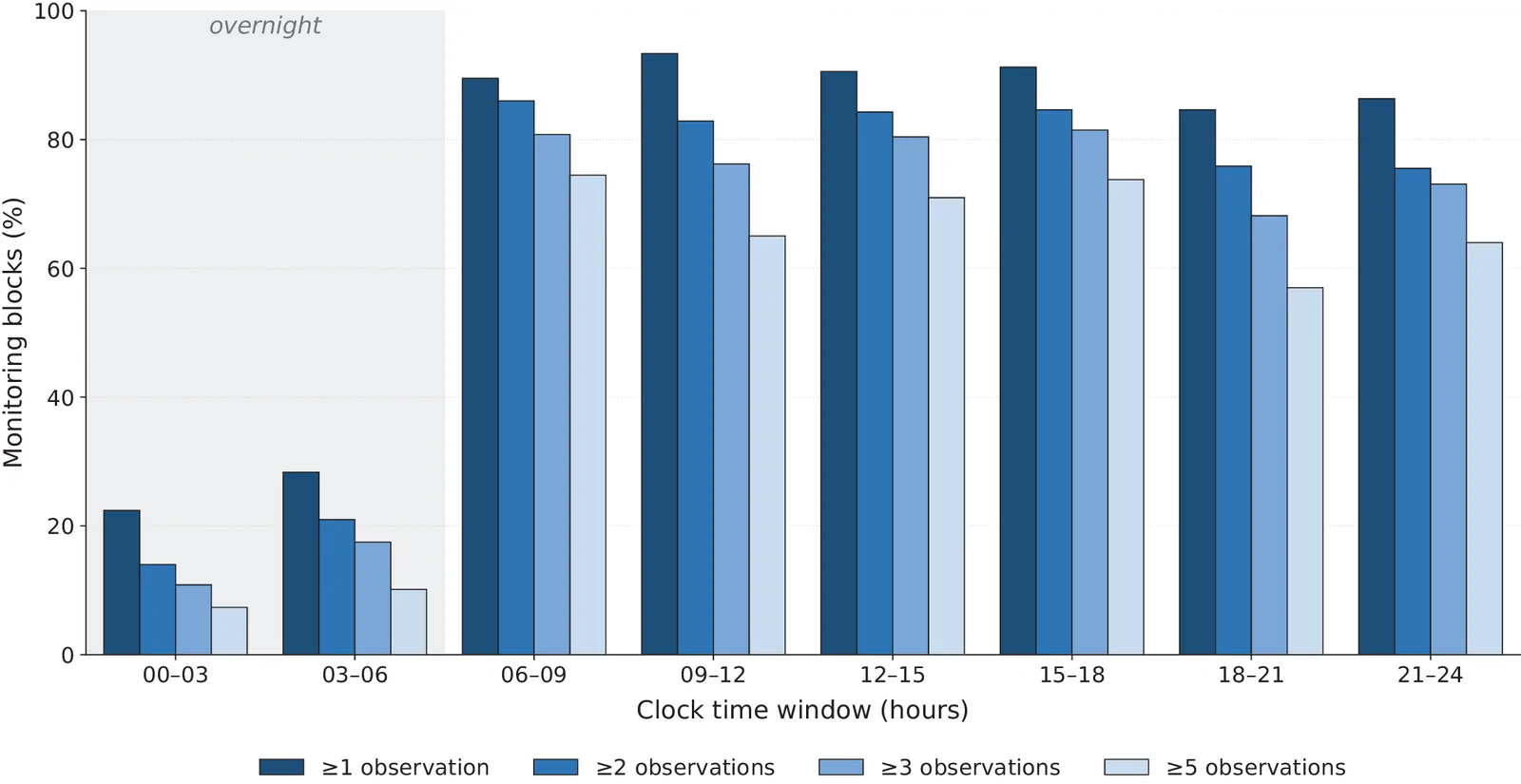
Distribution of iCare HOME self-measurements across the clock face, summarized in 8 non-overlapping 3-hour windows across all 286 monitoring blocks. *Bars* show the percentage of monitoring blocks in which a given window contained at least one, at least two, at least three, and at least five observations, so that both the breadth and the depth of clock-face coverage may be judged. The *shaded* region marks the overnight windows from 00:00 to 06:00.

### Coverage Indices and Detection Class

Three mutually exclusive detection classes were defined. Class 1 comprised blocks with at least one of F1 to F4 characterized and no non-integer peak (*n* = 123), class 2 were those with at least one non-integer peak (*n* = 9, of which 5 also had a characterized integer harmonic), and class 3 had neither (*n* = 154). Kruskal–Wallis tests showed differences across classes on five of six per-block parameters (total observations H = 93.9, block duration H = 35.2, observations-per-window minimum H = 12.3, longest sparse gap H = 68.2, and clock-hour spread H = 78.2; all *P* ≤ 0.002), localized by Bonferroni-corrected pairwise tests to the class 1 versus class 3 contrast (adjusted *P* ≤ 0.001 on every metric). Class 1 had a median observation count of 103 (IQR = 76–129) against 40 (IQR = 11–81) in class 3, a median clock-hour spread of 14 (IQR = 12–17) versus 8 (IQR = 3–13), and a lighter upper tail on the longest sparse gap (IQR = 3–6 hours versus 6–12 hours). The observations-per-window minimum was zero-inflated (median 0 in every class) and residual root mean square (RMS) did not differ across classes (H = 0.24, *P* = 0.89; Fig. 5).

**Figure 5.**
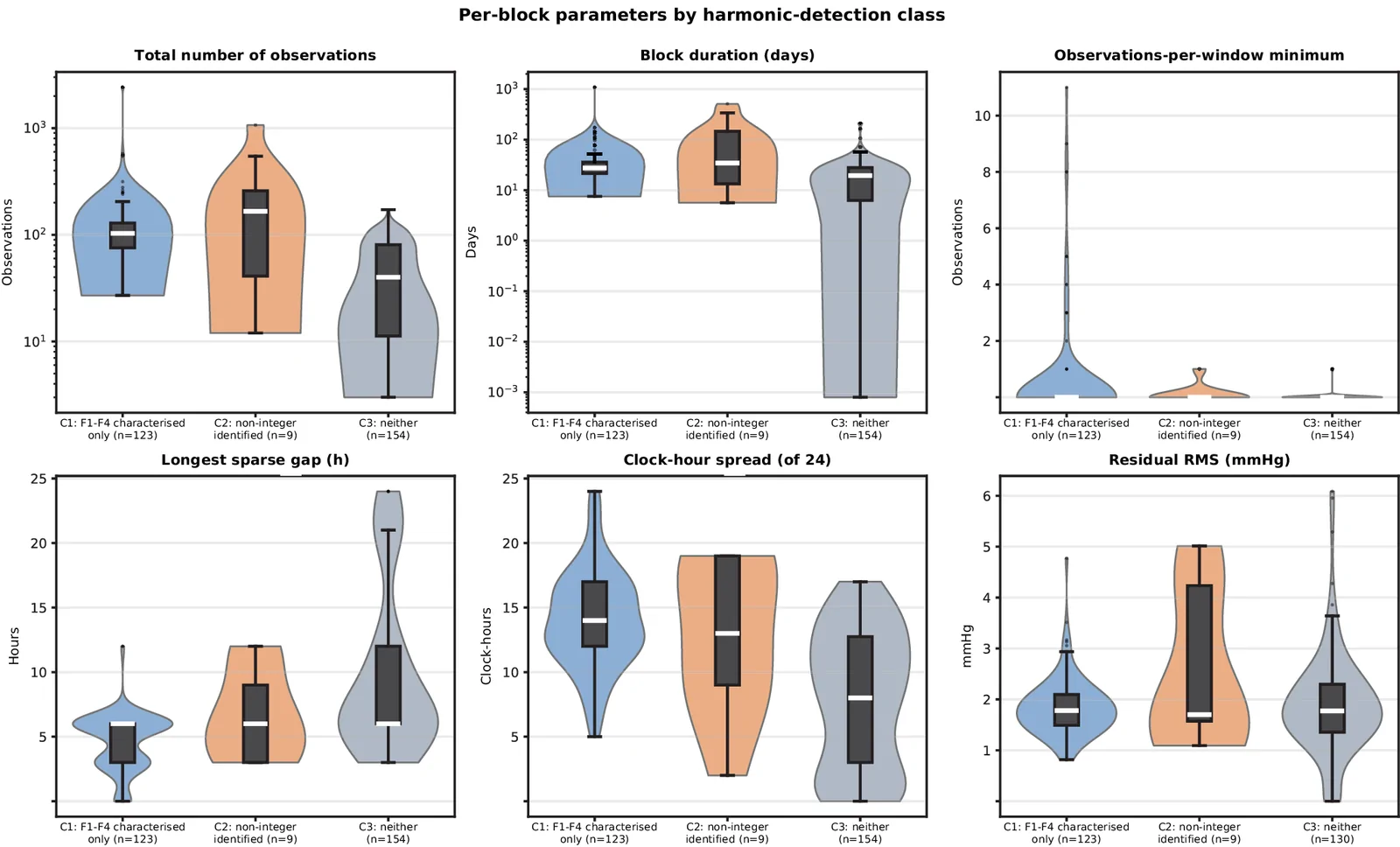
Distributions of six per-block parameters across the three harmonic-detection classes. Violins show the kernel-density estimate of each distribution, embedded boxes show the interquartile range, the *white line* shows the median and whiskers the 1.5 × IQR limits. Total observations and block duration are on log axes to accommodate heavy right skew. C1, in *blue*, are blocks with at least one of F1 to F4 characterized and no non-integer peak (*n* = 123). C2, in *orange*, are blocks carrying a non-integer Lomb–Scargle peak under the shared 5%-of-frequency rule (*n* = 9, including 5 blocks with both classifications). C3, in *grey*, are neither (*n* = 154; the residual RMS panel shows the 130 of these for which a harmonic fit was attempted).

### Detection by Observation-Count Stratum

Composite characterization rate rose steeply with the number of observations (Table 4), from 7.1% below 40 observations to 34.0% at 40 to 70, 55.2% at 70 to 100, 68.4% at 100 to 130, 78.6% at 130 to 160, and 96.0% above 160. The 70 to 100 stratum is the lowest at which more than half of the blocks yield a characterized harmonic. F2 characterization was at or below 2.4% under 70 observations and rose to 57.1% at 130 to 160; F3 and F4 were less common but followed a similar pattern.

**Table 4.** Per-Frequency and Composite (any of F1–F4) Characterization Rates Stratified by Total Observation Count (N_obs)

Observation-Count Stratum	*n*	Median Observations	F1 Char, %	F2 Char, %	F3 Char, %	F4 Char, %	Any F1–F4, %
<40	85	13	5.9	2.4	0.0	0.0	7.1
40–70	47	58	27.7	2.1	0.0	8.5	34.0
70–100	58	84	41.4	12.1	3.4	3.4	55.2
100–130	57	110	56.1	15.8	19.3	7.0	68.4
130–160	14	134	50.0	57.1	14.3	14.3	78.6
>160	25	248	84.0	56.0	24.0	16.0	96.0

Wider 95% binomial confidence intervals apply at the smaller strata, particularly the 130–160 stratum where *n* = 14.

### Joint Coverage–Gap Surface

To combine the coverage descriptors into a single model we fitted a logistic GAM with a two-dimensional tensor smooth on clock-hour spread and longest sparse gap plus a smooth on total observations (Supplementary Section S3). Figure 6 displays the predicted detection-probability surface at four representative observation counts (50, 100, 130, and 180), shown only inside the convex hull of supported data near each value. At 50 observations, no combination of coverage and gap tolerance exceeds 50% predicted probability. The longest sparse gap is the dominant constraint once a moderate clock-hour spread has been achieved, and the gap tolerated at a given probability depends on the total number of observations. At 100 observations, the 75% contour lies at a gap of approximately 3 hours. At 130 observations, it lies at approximately 6 hours, and the 90% contour is attained only at the widest clock-hour spread and a gap below approximately 1.5 hours. At 180 observations, the 95% contour is reached at a gap of approximately 3 hours. The contours in Figure 6 bend rather than run straight because the tensor smooth allows the two coverage measures to interact, so that the sparse gap tolerated at a given probability itself shifts as the clock-hour spread widens. Each value quoted is therefore read from the fitted surface at a stated pair of coverage coordinates (Supplementary Section S3).

**Figure 6.**
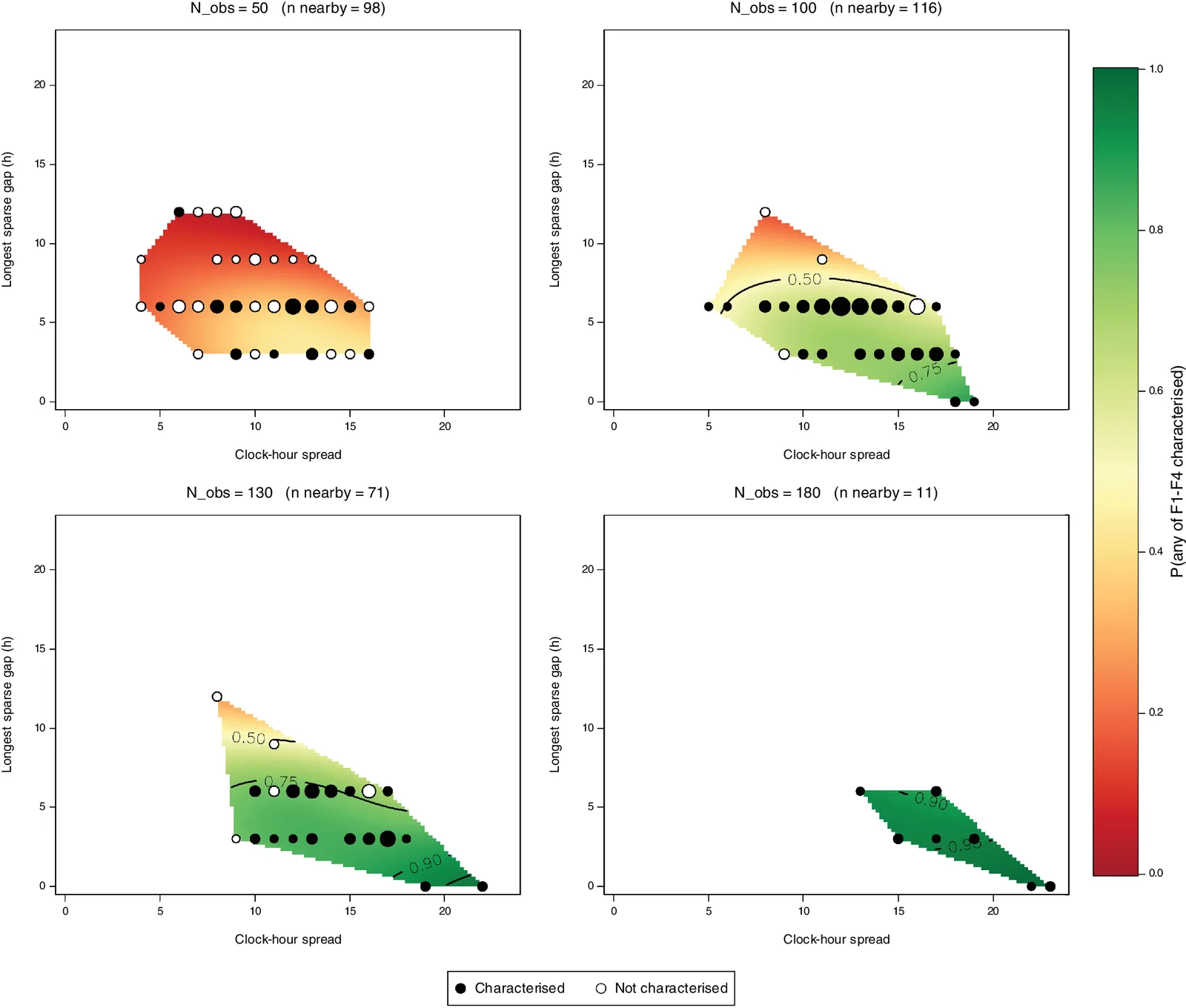
Tensor-product generalized additive model (GAM) showing P (any of F1–F4 characterized) as a joint function of the two coverage metrics, at four representative observation counts (50, 100, 130, and 180; panel titles use N_obs for the total number of observations). The clock-hour spread is the number of the 24 clock-hours containing at least 2 observations. The longest sparse gap is the longest unbroken run of clock-hours each containing one or no observation. The “n nearby” label above each panel gives the number of blocks whose observation count lies within ±30 of that panel’s target, so the 180-observation panel rests on only 11 blocks and should be interpreted accordingly. Heatmap color (*red* to *green*) encodes predicted probability from 0 to 1, with contours at 50%, 75%, 90%, and 95% read on the vertical (longest sparse gap) axis. *Circles* mark unique coverage coordinates, circle area scaling with the number of blocks there, and fill color indicating the majority class (*black* for characterized and *white* for not characterized). The surface is shown only inside the convex hull of observed values for the nearby subset, to avoid extrapolation. Where the longest sparse gap is approximately 6 hours the surface turns down slightly above a clock-hour spread of approximately 15, but that region rests on few blocks and is not interpreted as a real effect.

### Amplitudes and Phase Precision of Characterized Harmonics

When characterized, the 24-hour rhythm had a median amplitude of 1.75 mm Hg (IQR = 1.09–2.89) and a median acrophase 95% confidence interval of 2.00 hours (see Table 5); amplitudes are mean-to-maximum. Median amplitudes were 1.56, 1.15, and 1.33 mm Hg at the 12-hour, 8-hour, and 6-hour harmonics, with acrophase intervals of 0.83, 0.80, and 0.49 hours, the tightening reflecting the larger number of cycles within a typical block. The lower 95% confidence bound on amplitude exceeded 0.4 mm Hg for all 4 harmonics and 0.9 mm Hg for the 24-hour component. Phase was summarized by circular statistics on each harmonic’s own period, referenced to midnight (Supplementary Section S2). The 24-hour rhythm reached its maximum at 11.1 hours (circular SD = 3.9 hours), a late-morning acrophase, about which the per-block estimates clustered clearly (mean resultant length *R* = 0.60, against 0.10 expected under uniformity at this n; Rayleigh *P* < 0.001). The 12-hour harmonic peaked at 4.8 and 16.8 hours and was also clearly clustered (circular SD = 2.1 hours, *R* = 0.55 against 0.16 expected, *P* < 0.001), as was the 8-hour harmonic at 2.4, 10.4, and 18.4 hours (circular SD = 1.5 hours, *R* = 0.50 against 0.22 expected, *P* = 0.004). Only the 6-hour harmonic was unconcentrated, with *R* = 0.31 against 0.25 expected (*P* = 0.22). The 12-hour and 8-hour components therefore reach their maxima at reproducible clock times across the cohort, as would be expected if they largely describe the shape of a shared daily pattern rather than independent oscillations.

**Table 5.** Amplitude and Phase Summaries of the Four Most Commonly Characterized Harmonic IOP Rhythms

Frequency	*n* Char.	Amp. Median, mm Hg	Amp. IQR, mm Hg)	Lo-95% CI Median, mm Hg	Acrophase 95% CI Median, h	Acrophase Circ. Mean, h	Acrophase Circ. SD, h
Freq 1 (24 h)	102	1.75	1.09–2.89	0.94	2.00	11.1	3.89
Freq 2 (12 h)	41	1.56	0.95–2.43	0.81	0.83	4.8	2.08
Freq 3 (8 h)	21	1.15	0.83–1.49	0.49	0.80	2.4	1.49
Freq 4 (6 h)	16	1.33	1.04–1.55	0.76	0.49	0.7	1.47

Amplitude and 95% confidence interval columns are reported as medians and interquartile ranges across the characterized blocks. Phase summaries are computed using circular statistics because phase is a periodic quantity defined on the harmonic's own period. Acrophase is the clock time of the fitted maximum, referenced to midnight; harmonic k reaches that maximum again every 24/k hours, so the 12-hour harmonic also peaks 12 hours after the tabulated time, the 8-hour harmonic every 8 hours, and the 6-hour harmonic every 6 hours.

## Discussion

The four questions put at the outset can be answered directly. Multi-harmonic structure is common rather than exceptional, because 2 or more of F1 to F4 were characterized together in 15.0% of blocks and 38.2% of the blocks carrying a 24-hour rhythm carried a higher harmonic as well. The two methods differ, the multi-harmonic Fourier fit being the more sensitive at every harmonic and increasingly so at higher ones, because it tests a grid fixed in advance while the Lomb–Scargle scan pays a multiple-comparison cost across its whole window. The constituent periodicities lie overwhelmingly on integer multiples of the daily cycle, with 15 non-integer peaks in 286 blocks and only one frequency recurring in a second subject. In addition, the sampling required is within reach of a motivated patient, roughly 100 to 130 observations spread across the clock face. Each answer is developed below.

Two findings stand out. A 24-hour IOP rhythm is common in treated glaucoma without being universal, appearing in 35.7% of recording blocks, with at least one of the 4 lowest harmonics in 44.8%. More striking is how often the curve carries several frequencies at once. Cosinor rhythmometry, still the dominant method for IOP circadian data,^8^^–^^10^^,^^15^ cannot by construction see above the fundamental, and earlier dual-harmonic regressions^17^^,^^18^ gave no per-component confidence intervals, so this prevalence has not been quantified before in glaucoma.

Approximately 9% of blocks carried a higher harmonic with no characterized 24-hour fundamental at all, which is the more intriguing result. Higher harmonics cannot then be merely a way of describing a non-sinusoidal circadian curve, because, in these eyes, there was no resolvable circadian curve to describe. A component appearing alone at 12 or 8 hours is more readily explained by what a patient does at much the same time each day, with twice-daily topical dosing the obvious candidate, alongside sleep and waking transitions, meals, and posture change. Separating an endogenous ultradian rhythm from an entrained behavioral one will need recordings annotated with dosing and sleep times, which these are not.

Non-integer periodicities were rare. The LSP can find a rhythm anywhere in its scan window, yet returned only 15 non-integer peaks across 9 of 286 blocks. Re-fitting each of them alongside the integer harmonics of its own block, under the criteria applied to those harmonics, confirmed nine. What almost none of them did was recur. Applying the 5% rule to the peaks themselves, only one frequency was shared by 2 of them, 0.947 cycles per day in 2 subjects, and that sits 0.053 cycles per day from the 24-hour fundamental, only 0.003 cycles per day outside the window that would have made it F1. Every other confirmed peak lies at least a fifth away in frequency from the nearest other. The single apparent recurrence is most likely a near-miss on the daily rhythm rather than a periodicity of its own, and the remaining peaks are what resolution artefacts in short records and slow drift in long ones would likely produce (Supplementary Section S5). A periodicity seen once in one eye and never again is not evidence of a physiological rhythm, and that is the sense in which these detections remain unpersuasive. The integer grid, by contrast, is occupied repeatedly and across subjects. The Lomb–Scargle is therefore best read as a conservative independent check rather than a more sensitive alternative.

The spectral form we find, power confined to integer multiples of the daily cycle, is the form seen in ambulatory blood pressure, where a 24-hour rhythm is near-universal, a 12-hour harmonic is common, and no reproducible non-integer rhythm has been found.^20^^,^^21^^,^^32^ Cortisol behaves much the same way, whereas glucose carries a genuine sub-circadian oscillator near 80 to 140 minutes that sits off the daily grid altogether.^22^^,^^23^^,^^33^ The comparison is one of spectral form and not of mechanism, and its value is in showing which framework is appropriate for IOP modeling. It also opens a therapeutic question, because where a patient’s phase can be estimated, dosing might be timed against the predicted pressure peak in order to blunt it, as proposed in hypertension.^20^

Rather more than half of the blocks yielded no characterized harmonic, and it would be wrong to read that as an absence of rhythm. Most of those blocks lie in the lower observation-count strata, where the model predicts little chance of detection whatever the underlying physiology. A separate consideration is that we did not record whether a patient had undergone glaucoma drainage surgery, which tends to flatten diurnal pressure variation and would therefore leave less amplitude available for detection.

The unit of analysis is also the block rather than the patient, so we report the probability that a monitoring period of given sampling quality will reveal a rhythm, not the proportion of patients who possess one. Many patients contributed more than one block, and characterization may not be consistent between a patient’s own blocks: the same eye could yield a characterized rhythm in one monitoring period and not in another, for various reasons. including varying timing and numbers of measurements. as well as the possibility that rhythms vary with time. We do not report congruency between a patient’s blocks, because blocks were recorded under varying conditions in the absence of protocol instructions. Some rhythms may have been altered by treatment changes, which were not recorded.

The coverage analysis turns all this into something a clinic can act on. Where 15 or more clock-hours carry at least 2 observations and no more than 2 consecutive clock-hours carry one or none, roughly 100 to 130 self-measurements over 4 weeks give a predicted characterization probability of 75% to 85%, and approximately 150 lift that to 90%. Below roughly 50 observations no arrangement of measurement times reaches even 50%, so a short record that finds nothing tells us about the record rather than the patient. Once a reasonable clock-hour spread is reached, the longest sparse gap is what limits detection. A patient who cannot measure overnight cannot close that gap, but its cost can be offset by measuring more often during the hours that are available (Supplementary Section S6).

### Limitations

This is a convenience sample of treated clinic patients rather than a prospective cohort, so absolute detection rates may not generalize, although the shape of the sampling relationship should. The high-observation end of the coverage surface rests on few blocks and we did not stratify by severity or treatment. We also could not assess posture-driven variation,^4^ which may contribute to higher-harmonic structure, because non-sitting posture occurred in only 0.16% of posture-noted measurements. Two limitations follow from the method itself. It describes what recurs, and is blind to transient excursions that do not repeat at a consistent time of day. These may well matter to glaucoma progression and neither snapshot clinic tonometry nor this analysis captures them, so a harmonic description may complement rather than replace other measures of pressure variability. The fit also assumes amplitude and phase hold steady across a block, so a rhythm that drifts or waxes and wanes sums less coherently and is pushed toward non-detection, a conservative failure that makes the rates reported here lower bounds.

## Conclusions

Multi-harmonic structure in the diurnal IOP curve is present in a substantial minority of treated patients with glaucoma, and the frequencies carrying real power lie almost entirely on integer multiples of the daily cycle. Modeling that curve as a single 24-hour cosine therefore misses something real in a clinically meaningful fraction of patients. Self-tonometry can resolve the structure with a protocol patients can keep to, roughly 100 to 130 observations over 4 weeks spread so that at least 15 of the 24 clock-hours carry 2 or more and no more than 2 consecutive clock-hours carry one or none, with approximately 150 observations lifting the chance of characterizing a rhythm to 90%.

## Supplementary Material

Supplement 1
