# Supplementary material for: Detecting Intraocular Pressure Harmonic Frequencies in Glaucoma and the Sampling Targets Needed to Identify Them With Self-Tonometry
Source: Invest Ophthalmol Vis Sci. 2026 Oct 21;67(12):37. doi: 10.1167/iovs.67.12.37 (PMC13712386; doi:10.1167/iovs.67.12.37)
Supplement: Supplementary file 1 [file iovs-67-12-37_s001.docx]

**Supplementary Material**

*Detecting intraocular pressure harmonic frequencies in glaucoma, and the sampling targets needed to identify them with self tonometry*

Morgan WH, Chiu C-N, Turpin A, Obreschkow D, McKendrick AM

This document provides the methodological detail relocated from the main Methods, together with an extended results passage on the non-integer detections and expanded sampling guidance. Section numbering (S1–S6) matches the citations in the main manuscript. Reference numbers are those of the main reference list; no new references are introduced here.

# **S1. Integer-harmonic acceptance windows**

The Fourier decomposition stage evaluates power on a grid of integer multiples of one cycle per day and applies a 5%-of-frequency tolerance so that a peak found at a slightly non-exact frequency may still be assigned to an integer harmonic. A frequency f is deemed to lie on integer harmonic k (in cycles per day) when |f − k| < 0.05 × k cycles per day. Table S1 tabulates the resulting absolute frequency tolerance and the equivalent tolerance expressed in period (hours) at each harmonic from F1 (24 h) to F10 (2.4 h). These windows are used to classify the integer-versus-non-integer status of both the Fourier peaks and the Lomb-Scargle peaks (Figure 3 and Table 3 of the main manuscript). It is worth stating explicitly what the window does and does not do. Power is never summed or integrated over the ±5% band. A peak inside the window is treated as evidence that the harmonic is present, and the amplitude and phase of that harmonic are then estimated by the joint least-squares re-fit at the exact integer frequency, which is what recovers the power carried by a group of neighbouring peaks in a single component. Nor is a peak inside the window discarded. A peak outside the windows of all ten harmonics takes no part in the integer-harmonic model at all, and is adjudicated only by the Lomb-Scargle scan, which is why a tall inter-harmonic feature can appear in a power spectrum without ever becoming a candidate for characterisation.

| **Harmonic** | **Period (h)** | **Frequency tolerance (± c/d)** | **Period tolerance (± h)** |
| --- | --- | --- | --- |
| **F1** | 24.0 | 0.05 | 1.20 |
| **F2** | 12.0 | 0.10 | 0.60 |
| **F3** | 8.0 | 0.15 | 0.40 |
| **F4** | 6.0 | 0.20 | 0.30 |
| **F5** | 4.8 | 0.25 | 0.24 |
| **F6** | 4.0 | 0.30 | 0.20 |
| **F7** | 3.4 | 0.35 | 0.17 |
| **F8** | 3.0 | 0.40 | 0.15 |
| **F9** | 2.7 | 0.45 | 0.13 |
| **F10** | 2.4 | 0.50 | 0.12 |

**Table S1.** The 5%-of-frequency acceptance windows for the ten integer harmonics. The frequency tolerance is 0.05·k cycles per day, where k is the integer harmonic number; the period tolerance is 24/(k ± tolerance), rounded to two decimal places.

## **S1.1 Block construction and the sparse-period filter**

A patient record was divided into break-separated blocks wherever ten or more days separated consecutive measurement days. Within a block a further density rule was applied. Any 14-day window containing fewer than 20 observations was marked sparse, and days lying inside such a window that carried two or fewer observations were removed. The observation counts reported throughout the manuscript are counts of the series that survives this filter.

## **S1.2 The noise threshold, and what is and is not simulated**

The Fourier decomposition stage retains a frequency when its power is too large to be explained by measurement variability and uneven sampling alone. The reference against which power is judged is analytic rather than simulated. Writing N for the number of observations in a block and s² for the variance of the measured pressures, the power returned at any frequency by a series of uncorrelated measurements follows an exponential distribution with mean Ns². That mean is the noise level used throughout. It is the same at every frequency, because it depends only on how many measurements were taken and how variable they were, and not on the periodicity being tested. Both the observed power and the reference level are ordinates of the unnormalised periodogram at one frequency, |Σ(y − ȳ)exp(−2πift)|², and not spectral densities, so both carry units of mmHg² and their ratio is dimensionless. Ns² is the power expected at a single tested frequency and not the power integrated across the spectrum, so no division by a frequency interval is required. Had both quantities instead been expressed as densities in mmHg² per cycle per day, each would have been divided by the same bandwidth and the ratio, and therefore every result reported here, would be unchanged.

Observed power P is expressed as the ratio S = P / N*s*². Under the null hypothesis the probability of a ratio at least as large as S is exp(−S), so the quantity reported in the analysis output as the noise threshold is 1 − exp(−S). A frequency is retained when the noise threshold exceeds 0.999, which is the same as requiring S to exceed −ln(0.001) = 6.91. The 90th, 99th and 99.9th centiles of the null distribution, drawn as nested grey bands in main manuscript Figure 1, lie at S = 2.30, 4.61 and 6.91 respectively.

Three properties of this test are worth stating. Power is evaluated at the true irregular observation times, so no interpolation, binning or resampling onto a uniform mesh occurs at any point. The test is applied frequency by frequency and carries no correction for the number of frequencies examined, which is one reason a grid of 10 integer harmonics fixed in advance is preferred here to a continuous scan, since the scan pays a multiple-comparison cost that the grid does not. And because the test is analytic, the Fourier detection counts are exactly reproducible, a repeat run on unchanged data returning identical numbers.

The only stochastic element anywhere in the analysis is the case-resampling bootstrap used for the amplitude and phase confidence intervals (Section S3.1). Because that bootstrap draws a finite number of replicates, a repeat run can move a small number of characterised classifications, of the order of one per cent of the frequency by block cells, without altering any conclusion.

# **S2. Lomb–Scargle scan and phase summary**

As an independent scan of the frequency axis, the generalised Lomb-Scargle periodogram^24–26,28^ was computed on each block using the *lomb* package in R, with the false-alarm probability calibrated by the method of Baluev.^29^ Because the periodogram fits a sinusoid to the actual measurement times rather than to interpolated values, it does not require regular spacing and the classical Nyquist constraint does not apply.^27^

The Lomb-Scargle search ran from 0.1 to 10.5 cycles per day, that is from a period of 10 days down to a period of 2.3 hours, so that it extended half a harmonic beyond F10 at its upper end and well below F1 at its lower end, allowing detection of both integer harmonics of the 24-hour fundamental and non-integer periodicities. For the integer-versus-non-integer classification of Lomb-Scargle peaks, the same 5%-of-frequency tolerance defined in Section S1 was applied, so that a peak was deemed to lie at integer harmonic k when |f − k| < 0.05 × k cycles per day. Applying the same rule across both methods makes their integer-harmonic counts directly comparable. Peaks returned by the full-range scan whose frequency lay outside the ±5% windows around all of F1–F10 were treated as non-integer detections. The full-range scan was computed at an oversampling factor of 10, while each integer harmonic was in addition scanned in its own ±5% window at an oversampling factor of 50, which is the value reported for that harmonic. A non-integer feature must therefore exceed the significance level of the whole scan, whereas an integer harmonic is judged within a window fixed in advance, and this difference in multiple-comparison cost, rather than any difference in how peak height is measured, is why a non-integer peak can be taller than an integer one and still not be confirmed.

## **S2.1 What Lomb-Scargle confirmation of an integer harmonic requires**

A significant Lomb-Scargle peak establishes that a periodicity was detected at that frequency, at a Baluev false-alarm probability below 0.001. Detection alone was not sufficient for an integer harmonic to enter the Lomb-Scargle column of main manuscript Table 2. That column applies three criteria jointly. The false-alarm probability at the harmonic must be below 0.001, the bootstrap lower 95% confidence bound on the amplitude of that harmonic must exceed zero, and the 95% confidence interval on phase must be narrower than 1/(2f) of a cycle at frequency f cycles per day, which for integer harmonic k is an acrophase interval narrower than 24/(2k²) hours (Section S3.1). The amplitude and phase quantities are those obtained from the joint least-squares refinement at the exact integer frequency, which are the same estimates that define Fourier characterisation. The Lomb-Scargle and characterised columns are therefore like-for-like, since both require amplitude and phase to be resolved to the same precision and they differ in how detection was established, by the noise-threshold test in the Fourier arm and by the independent periodogram's false-alarm probability in the Lomb-Scargle arm. Applying the amplitude and phase criteria reduces the raw count of false-alarm-probability-significant integer peaks across the 286 break-separated blocks from 99, 49, 24 and 27 at F1–F4 to 91, 32, 10 and 5, and from between 4 and 13 at each of F5–F10 to at most one. The raw counts are not reported in the main manuscript because, resting on detection alone, they are not comparable with the characterised column. Non-integer peaks lie off the integer grid, so no refinement applies to them and they are reported on false-alarm probability alone.

## **S2.2 Joint refit of the non-integer peaks**

Nothing in the least-squares formulation requires a frequency to be an integer multiple of one cycle per day. The design matrix is evaluated at the true observation times at whatever frequencies are supplied, so a non-integer peak can be refitted in exactly the way an integer harmonic is. Each of the 15 non-integer Lomb-Scargle peaks was therefore added to the set of Fourier-significant integer harmonics of its own block and the whole set refitted in one model, with amplitude and phase confidence intervals from a 2,000-replicate case-resampling bootstrap, twice the 1,000 replicates used for the integer harmonics (Section S3.1), because these frequencies lie off the fitted grid and their intervals are the quantity on which confirmation turns. The criteria applied were those used for the integer harmonics, a bootstrap lower 95% confidence bound on amplitude above zero and a 95% confidence interval on phase narrower than 1/(2f) of a cycle. Nine of the 15 met both criteria.

Three cautions attach to that count. First, the amplitude criterion passed for all 15 peaks and carries little information here, because each frequency was selected as the largest peak in a continuous scan, so testing the amplitude of the selected frequency is post-selection inference and the bootstrap interval makes no allowance for the selection. The phase criterion is the one that separates the peaks. Second, below 0.5 cycles per day the permitted phase interval, 1/(2f) of a cycle, exceeds a whole cycle, so the phase criterion cannot fail at the two confirmed peaks whose period exceeds 48 hours, at 0.347 and 0.463 c/day. Third, one confirmed peak, SUBJ01 at 3.417 c/day, has a variance inflation factor of 8.7 against the integer harmonics fitted in the same 41-observation block, meaning the record is not long enough to separate it from its neighbours. Excluding those three, SUBJ03 at 0.347 c/day, SUBJ02 at 0.463 c/day and SUBJ01 at 3.417 c/day, leaves six peaks confirmed on a criterion that carries force. Among those six, one frequency still recurs, the 0.947 c/day peak carried by both SUBJ04 and SUBJ05, and that lies 0.003 c/day outside the window that would have made it F1. No other frequency appears in more than one subject.

## **Phase summary by circular statistics**

The refinement stage fits a mean level plus one cosine per retained harmonic, A cos(2πkt/24 + φ) for harmonic k, with t measured in hours from midnight. For a single harmonic this is exactly the cosinor model of Nelson and colleagues,^15,16^ and the multi-harmonic fit is its natural generalisation, all retained harmonics being estimated together in one least-squares solution rather than one at a time. Phase is reported as the acrophase, the clock time at which the fitted component reaches its maximum, referenced to midnight. Because harmonic k completes k cycles in 24 hours, its acrophase repeats every 24/k hours, so the tabulated value for the 12-hour harmonic is the earlier of its two daily maxima, that for the 8-hour harmonic the earliest of three, and that for the 6-hour harmonic the earliest of four. Phase summaries are computed with circular statistics because phase is a periodic quantity defined on the harmonic's own period P (24 h for F1, 12 h for F2, 8 h for F3, 6 h for F4). Each acrophase is wrapped into the interval [0, P) and converted to an angle on the unit circle. The circular mean is recovered from the resultant vector and reported in hours on that period. The circular standard deviation is √(−2·ln R)·P/(2π), where R is the length of the mean resultant vector. R takes a value between 0, when the per-block acrophases are spread evenly around the clock, and 1, when they coincide. The expected value of R under a null hypothesis of uniform phase depends on sample size. The reference values for n = 102, 41, 21 and 16 (the F1–F4 characterised counts) are 0.10, 0.16, 0.22 and 0.25 respectively, and are the values against which the empirical R is compared in the main text, where the Rayleigh test of uniformity is also reported.

# **S3. Confidence intervals and the coverage–detection model**

## **S3.1 Bootstrap confidence intervals for amplitude and phase**

Because absolute amplitude is constrained to be non-negative and therefore has a right-skewed sampling distribution, analytical (normal-theory) confidence intervals on amplitude and phase are unreliable. Interval estimates were instead obtained by a non-parametric case-resampling bootstrap with 1,000 replicates. The joint refit of the non-integer peaks described in Section S2.2 used 2,000 replicates; 1,000 is the figure for every integer-harmonic interval reported in the main manuscript. Within each block the observed (time, IOP) pairs were resampled with replacement to the original block size; the multi-harmonic model was refitted to each resampled set at the same fixed integer frequencies retained for that block, at the true irregular observation times and with no interpolation; and the amplitude and phase of each retained harmonic were recorded. The 2.5th and 97.5th percentiles of the 1,000 bootstrap estimates were taken as the 95% confidence interval for each parameter (the percentile method). Phase intervals were computed on the circular scale of the harmonic's own period to respect wrap-around.

The bootstrap interval enters the characterisation rule directly. A frequency was classified as characterised only when all three criteria were met. These were noise-threshold significance at the Fourier decomposition stage, a bootstrap-validated lower 95% confidence bound on amplitude greater than zero, and a 95% confidence interval on phase narrower than 1/(2f) of a cycle at frequency f cycles per day, which for integer harmonic k is an acrophase interval narrower than 24/(2k²) hours. Expressing the criterion as a fraction of a cycle has two consequences. It applies unchanged to the non-integer frequencies refitted in Section S2.2, and it requires phase precision in proportion to how often a component recurs within the day, so that a component claimed to repeat four times daily must have its timing pinned down four times as tightly as the 24-hour fundamental. The rule is therefore more demanding at higher harmonics than a flat half-cycle criterion, which would permit an acrophase interval of 24/(2k) hours. That flat rule would raise the characterised counts at F2, F3 and F4 from 41, 21 and 16 to 47, 25 and 22, and the total across all ten harmonics from 185 to 208. The counts reported throughout this paper are on the stricter rule and are therefore conservative.

**Equivalence to a least-squares periodogram, and the Nyquist limit.** The refinement fit is a least-squares solution of the complex-exponential design matrix evaluated at the true irregular observation times, without resampling or interpolation. It is in that sense equivalent to the least-squares formulation of the Lomb-Scargle analysis used at the next stage.^24–28^ Because neither procedure requires a uniform sampling interval, the classical Nyquist constraint does not apply directly. The practical upper frequency limit is set instead by cumulative phase coverage, meaning how completely the observations, folded onto the period of a candidate harmonic, fill that period. This is the reason the coverage metrics of Section S3.2 are defined on the clock face rather than along calendar time.

## **S3.2 Generalised additive model specification and comparison**

The probability of characterising at least one of the four most common integer harmonics (F1, F2, F3 or F4) was modelled by a logistic generalised additive model (GAM). A GAM was preferred to a parametric logistic regression because the coverage predictors departed strongly from normality. The total number of observations and the block duration were heavily right-skewed (Shapiro-Wilk *p* < 10−21 in the characterised subset), the observations-per-window minimum was zero-inflated (median, first and third quartiles all zero), and the coverage and gap metrics showed bounded discretisation arising from the integer-hour quantisation of the clock-hour data. The penalised smooth terms of a GAM accommodate such predictors without distributional assumptions and let the data determine the functional form of the predictor–outcome relationship. Smoothing penalties were selected automatically by restricted maximum likelihood (REML).

Three specifications were compared, using the variable names of the analysis dataset, in which Hours_With_2Plus_Obs is the clock-hour spread, LargestGap_leq1obs_hours the longest sparse gap and N_Observations the total number of observations.

**Model A, joint tensor-plus-smooth**

characterised ~ te(Hours_With_2Plus_Obs, LargestGap_leq1obs_hours, k = c(5,5)) + s(N_Observations, k = 6)

**Model B, tensor alone**

characterised ~ te(Hours_With_2Plus_Obs, LargestGap_leq1obs_hours, k = c(5,5))

**Model C, tensor with categorical observation-count stratum**

characterised ~ te(Hours_With_2Plus_Obs, LargestGap_leq1obs_hours, k = c(5,5)) + N_obs_stratum

| **Model** | **AIC** | **Pseudo-R²** |
| --- | --- | --- |
| **A — joint tensor-plus-smooth** | **266.4** | **0.35** |
| B — tensor alone | 291.7 | 0.28 |
| C — tensor + categorical readings-count | 281.9 | 0.32 |

**Table S2.** AIC and pseudo-R² for the three candidate GAM specifications. Model A had the lowest AIC and highest pseudo-R² and is the specification reported in the main manuscript.

The chosen model was implemented in the *mgcv* package in R^30,31^ with the binomial family and REML smoothness selection. The two-dimensional tensor-product smooth jointly fits the clock-hour spread and the longest sparse gap on a 5 × 5 basis, allowing local interaction between them, while the one-dimensional smooth captures the additional effect of the total number of observations. The prediction surface is rendered as model-predicted detection probability over the coverage–gap plane with contours at the 50%, 75%, 90% and 95% thresholds, displayed only inside the convex hull of the observed predictor combinations to avoid extrapolation (main manuscript Figure 6).

**Reading the surface.** Three cautions apply. First, the two coverage predictors are necessarily anti-correlated, since a block whose observations are spread across many clock-hours cannot also carry a long run of sparse clock-hours, so the joint surface should be read only within the regions of the plane that the data actually populate. That is why the plotted surface is clipped to the convex hull. Second, panel support is very uneven, with 98, 116, 71 and 11 blocks lying within ±30 observations of the four panel targets, so the 180-observation panel is the least well determined. Third, the iso-probability contours are curved rather than straight, because the tensor smooth allows the two coverage measures to interact, so the sparse gap tolerated at a given probability itself shifts as the clock-hour spread widens. Every probability quoted in the main manuscript is therefore read from the fitted surface at a stated pair of coverage coordinates, not interpolated along a contour treated as a straight line. One consequence of the curvature is visible in the 130-observation panel, where at a longest sparse gap of about 6 hours the fitted surface turns down slightly as the clock-hour spread rises beyond about 15. That corner rests on few blocks, 3 of 7 characterised at a spread of 16 and 1 of 2 at a spread of 15, and is not interpreted as a real effect of wider clock-hour coverage.

# **S4. Justification of the non-parametric tests**

Per-block coverage and residual parameters (the total number of observations, block duration, observations-per-window minimum, longest sparse gap, clock-hour spread and residual RMS) were compared between the three harmonic-detection classes by a procedure appropriate to their distributions. For each parameter the Kruskal–Wallis test served as the omnibus comparison across the three classes; where it was significant, pairwise Mann–Whitney U tests with Bonferroni correction for three comparisons localised the difference. Non-parametric tests were chosen because every parameter failed a Shapiro-Wilk test of normality within at least one class, and because the class sizes are markedly unequal (n = 123, 9 and 154), which makes the assumptions of parametric alternatives untenable.

McNemar's test (continuity-corrected) compared paired Fourier and Lomb-Scargle detections at each integer harmonic, with Cohen's kappa reporting agreement. Because Lomb-Scargle confirmation applies the same amplitude and phase precision criteria as Fourier characterisation (Section S2.1), these are equivalent endpoints; the main manuscript additionally reports the comparison at the detection stage alone, in the Results under 'Detection rates across the harmonic frequency series and comparison of techniques', Fourier noise-threshold significance against Lomb-Scargle false-alarm probability. Median and interquartile range were used to describe every coverage metric because their non-normal shape makes the mean unrepresentative; Shapiro-Wilk tests confirmed the departure from normality in each class. Significance is reported at p < 0.05. All analyses were performed in R version 4.3.^30^

# **S5. Non-integer detections and residual structure**

## **Per-subject distribution of the non-integer peaks**

When the 15 non-integer peaks (main manuscript Table 3) were examined in relation to the integer-harmonic landmarks, four lay close to an integer (within 0.20 cycles per day) but outside the harmonic-specific 5% acceptance window. Two were near-identical F1 near-misses at 0.95 c/d contributed by *different* subjects, SUBJ04 (period 25.34 h) and SUBJ05 (period 25.35 h), each just outside the F1 ±0.05 c/d window. The third was an F1 near-miss at 1.17 c/d from SUBJ03 (period 20.57 h, 0.17 c/d from F1) and the fourth an F2 near-miss at 1.81 c/d from SUBJ01 (period 13.29 h, 0.20 c/d from F2). These near-miss peaks reflect the lower limit of frequency resolution achievable with the Lomb-Scargle periodogram on records of moderate length and may represent integer harmonics displaced by small numerical perturbations in peak detection.

The remaining eleven peaks were more substantially offset from any integer harmonic. SUBJ02 contributed four (at 0.46, 2.42, 7.35 and 9.44 c/d; periods 51.9, 9.9, 3.26 and 2.54 h), SUBJ01 contributed three further peaks at 2.80, 3.42 and 5.62 c/d (periods 8.59, 7.02 and 4.27 h), SUBJ03 contributed two long-period peaks at 0.35 and 1.42 c/d (periods 69.2 and 16.9 h), and SUBJ04 and SUBJ06 each contributed one substantially-offset peak (at 6.59 c/d and 8.54 c/d respectively). These offsets arose by two distinct routes. The SUBJ03 peaks come from recordings spanning 146 and 337 days, within which the Lomb-Scargle scan can resolve slow drift below the integer grid. The 69.2-h peak almost certainly reflects an unsampled multi-day interval within that block rather than a genuine 2.9-day physiological rhythm, and the 51.9-h peak from a 34-day SUBJ02 block has a similar interpretation. The other route is short, sparse records. The 25.34-h near-miss attributed to SUBJ04 comes from a block of only 12 observations across 5.6 days, at the limit of the frequency resolution the periodogram can support. There was no clustering of non-integer peaks at any specific fractional period that would indicate a distinct rhythm beyond the integer harmonics described in the main manuscript.

Grouping the peaks by the outcome of the joint refit (main manuscript Table 3) cuts across this description rather than following it. The nine confirmed peaks include both of the 0.947 c/day F1 near-misses, the long-period peaks at 0.347 and 0.463 c/day, the SUBJ03 peaks at 1.167 and 1.423 c/day, SUBJ02 at 2.421 c/day, SUBJ01 at 3.417 c/day and SUBJ04 at 6.590 c/day. The six that were not confirmed lie mostly at the high-frequency end, at 1.805, 2.795, 5.622, 7.351, 8.545 and 9.439 c/day, where a block of moderate length cannot pin the phase to within half a period. Confirmation therefore tracks record length and frequency rather than any physiological grouping, which is the pattern expected of resolution artefacts and slow drift rather than of a rhythm.

## **Do the non-integer peaks share frequencies with each other?**

The integer classification asks whether a peak lies within 5% of an integer multiple of one cycle per day. The same rule can be turned on the non-integer peaks themselves to ask whether any two of them share a frequency, which is what a genuine non-integer rhythm would require. Across all 15 peaks, and across the 9 confirmed peaks, exactly one pair falls within 5% of each other. SUBJ04 and SUBJ05 each carry a confirmed peak at 0.947 cycles per day, and the two agree to 0.07%. Matching on period rather than frequency gives the same single pair.

That one recurrence is a boundary case on the daily harmonic rather than a new periodicity. The frequency lies 0.053 cycles per day from F1, only 0.003 cycles per day outside the 0.05 acceptance window, so a slightly wider window would have classified both peaks as the 24-hour harmonic. The two blocks also differ greatly in what they can resolve. The SUBJ05 block spans 45.5 days, giving a Rayleigh frequency resolution of 0.022 cycles per day, so the 0.053 offset is about 2.4 resolution elements and is at least measurable. The SUBJ04 block spans 5.6 days and carries 12 observations, giving a resolution of 0.177 cycles per day, so the offset is 0.3 of a resolution element and the peak cannot be separated from F1 at all.

Every other confirmed peak is far from its neighbours. The smallest separation among the remaining seven is 19.8%, between the SUBJ03 peaks at 1.167 and 1.423 cycles per day, and those come from the two eyes of the same subject. The next smallest are 28.6% and 34.1%, both between different subjects. No non-integer frequency other than 0.947 cycles per day appears in more than one block.

## **Residual-RMS structure across detection classes**

Class 2 (the 9 blocks carrying a non-integer peak under the shared 5%-of-frequency rule, of which 5 also have at least one characterised integer harmonic) sits in an intermediate position on most coverage axes, with a median of 166 observations [IQR 41–258], closer to Class 1 than to Class 3 at the centre but with an IQR spanning both. The same intermediate position is seen on block duration and on the longest sparse gap. With nine blocks the class is too small to support formal inference, and the description below is offered as such.

The medians of the three residual distributions span a narrow range (Class 1, 1.78 mmHg [1.49–2.10]; Class 2, 1.70 mmHg [1.57–4.24]; Class 3, 1.77 mmHg [1.35–2.30], computed on the 130 of 154 Class 3 blocks for which a harmonic fit was attempted), but the Class 2 IQR is more than twice as wide as either of the others. The Kruskal–Wallis test on residual RMS across the three classes did not reach significance (H = 0.24, *p* = 0.89), and no pairwise comparison approached significance after correction. The visual bimodality of the Class 2 residuals is therefore a descriptive observation that, with only nine contributing blocks, we cannot distinguish statistically from chance. With that caveat, the qualitative pattern is consistent with two routes by which non-integer peaks arise, namely slow drift in well-sampled long-duration records (the long-period peaks at 0.35–1.42 c/d in main manuscript Figure 3) and sampling noise in short or sparse records (the more scattered peaks at higher cycles per day).

Two conclusions follow. First, the clock-hour spread and the upper tail of the longest sparse gap separate blocks with characterised harmonics from blocks without, with strong statistical support (all Bonferroni-adjusted *p* ≤ 0.001), which is why they are the parameters carried into the sampling recommendations. Second, blocks producing a non-integer Lomb-Scargle peak are not statistically distinguishable from the F1–F4-characterised blocks on any coverage or residual measure tested, supporting the interpretation that the few non-integer detections are not a separate phenomenon but the upper tail of the same distribution of well-sampled records that yields integer-harmonic characterisations.

# **S6. Expanded sampling guidance**

The main manuscript states the sampling recommendation in its shortest usable form. This section sets out the underlying trade-off, so that a protocol can be adapted to what a given patient can realistically achieve.

**The three axes.** Detection depends on three things that act together rather than independently. The total number of observations sets the headline probability. The clock-hour spread, meaning how many of the 24 clock-hours carry at least two observations, determines whether the phase of a harmonic can be resolved at all, because a harmonic of period P can only be identified if the observations, folded onto P, fill enough of that period. The longest sparse gap, the longest unbroken run of clock-hours carrying one or no observation, controls the upper tail of detection failure, since a single wide blind interval can defeat an otherwise generous record.

**Targets.** Where 15 or more clock-hours carry at least two observations and no more than two consecutive clock-hours carry one or none, the fitted model gives a probability of characterising at least one of F1–F4 of approximately 0.47 at 50 observations, 0.74 at 100, 0.85 at 130, 0.90 at 150 and 0.95 at 180. Widening the clock-hour spread to 19 under the same gap condition raises these to approximately 0.54, 0.79, 0.88, 0.92 and 0.96. Four weeks of monitoring at three to four observations per day reaches the 100–130 range; five to six weeks, or a higher daily rate, is needed for 150.

**Trading gap against count.** Once a moderate clock-hour spread has been achieved, the longest sparse gap becomes the main constraint, and the gap that can be tolerated depends on how many observations are taken. At a clock-hour spread of 15, a 75% probability survives a sparse gap of about 5.8 hours at 130 observations, against about 1 hour at 100. Put the other way, with an unavoidable eight-hour overnight gap at that spread the predicted probability rises from 0.39 at 100 observations to 0.57 at 130, 0.68 at 150 and 0.81 at 180. This is the practically useful form of the result for a patient who cannot measure overnight, since a gap that cannot be closed can still be paid for by measuring more often during the hours that are available.

**A floor.** Below approximately 50 observations no distribution of measurement times reaches even 50% predicted probability. A short or sparse record that yields no characterised harmonic is therefore uninformative about whether the patient has a rhythm, and should not be reported as a negative finding.

**A practical protocol.** Three to four observations per day for four to six weeks, deliberately rotated across the waking day rather than taken at the same clock times each day, with at least one late-evening and one early-morning observation where the patient is able, will in most cases satisfy both coverage conditions. Rotation matters more than regularity. A fixed twice-daily schedule at the same two clock times accumulates observations without accumulating clock-hour spread, and will not support harmonic characterisation however long it is continued.
